# The prevalence of redundant nerve roots in standing positional MRI decreases by half in supine and almost to zero in flexed seated position: a retrospective cross-sectional cohort study

**DOI:** 10.1007/s00234-022-03047-z

**Published:** 2022-09-09

**Authors:** Luca Papavero, Nawar Ali, Kathrin Schawjinski, Annette Holtdirk, Rainer Maas, Stella Ebert

**Affiliations:** 1 Clinic for Spine Surgery, Schoen-Clinic Hamburg, Academic Hospital of the University Medical Center Eppendorf, Dehnhaide 120, 22081 Hamburg, Germany; 2 CRO - Dr. med. Kottmann GmbH & Co. KG, Hamm, Germany; 3 Radiological Office Raboisen 38, Hamburg, Germany; 4 MRI-Center – Europa Passage, Hamburg, Germany

**Keywords:** Redundant nerve roots, Spinal stenosis, Positional magnetic resonance imaging, Posture, Lumbar spine

## Abstract

**Purpose:**

This retrospective cross-sectional cohort study investigated the influence of posture on lordosis (LL), length of the spinal canal (LSC), anteroposterior diameter (APD L1-L5), dural cross-sectional area (DCSA) of the lumbar spinal canal, and the prevalence of redundant nerve roots (RNR) using positional magnetic resonance imaging (MRI) (0.6 T).

**Methods:**

Sixty-eight patients with single-level degenerative central lumbar spinal stenosis (cLSS) presenting with RNR in the standing position (STA) were also investigated in supine (SUP) or neutral seated (SIT) and flexed seated (FLEX) positions. Additionally, 45 patients complaining of back pain and without MRI evidence of LSS were evaluated. Statistical significance was set at *p* < 0.05.

**Results:**

Controls (A) and patients with cLSS (B) were comparable in terms of mean age (*p* = 0.88) and sex (*p* = 0.22). The progressive transition from STA to FLEX led to a comparable decrease in LL (*p* = 0.97), an increase in LSC (*p* = 0.80), and an increase in APD L1-L5 (*p* = 0.78). The APD of the stenotic level increased disproportionally between the different postures, up to 67% in FLEX compared to 29% in adjacent non-stenotic levels (*p* < 0.001). Therefore, the prevalence of RNR decreased to 49, 26, and 4% in SUP, SIT, and FLEX, respectively.

**Conclusion:**

The prevalence of RNR in standing position was underestimated by half in supine position. Body postures modified LL, LSC, and APD similarly in patients and controls. Stenotic levels compensated for insufficient intraspinal volume with a disproportionate enlargement when switching from the STA to FLEX.

## Introduction

Experimental cadaver studies [1, 2], myelographic and CT-myelographic investigations [3], and positional MRI studies [4–10] have shown that posture influences lumbar lordosis (LL), length of the spinal canal (LSC), segmental anteroposterior diameter (APD), and dural cross-sectional area (DCSA) of the lumbar spine. Transitioning from the extended spinal canal (standing) to the inflected (bent forward sitting) decreases the lordotic angle, increases the length of the lumbar spine, and increases the capacitance of the spinal canal.

**Fig. 1:**
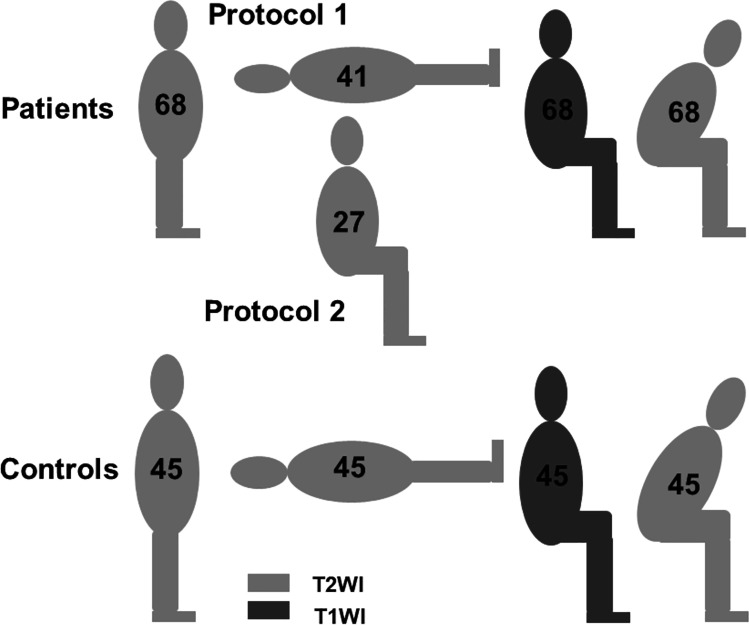
(Top) Study protocols of patients and (bottom) of controls

However, additional parameters are required to diagnose patients with degenerative central lumbar spinal stenosis (cLSS) presenting with redundant nerve roots (RNR) confirmed by MRI. First, we investigated the influence of four relevant postures on daily living. These are as follows: STA, which increases pain when lasts a long time; SUP, which during sleep is associated with 71% of patients affected by cLSS with nocturnal cramps [11]; SIT, which relieves the pain partially; and FLEX, which relieves pain as much as possible. Furthermore, psoas relaxed SUP is the standard positioning for MRI investigations due to lumbar stenosis. Second, we analyzed the influence of postural changes on the prevalence of RNR (Fig. 1).

RNR are coiled, elongated, and thickened cauda nerve roots (CNR) associated with severe stenosis [12]. In conventional supine MRI, the CNR are stretched on one side of the stenotic level and serpentine or loop-shaped on the other. Frequently, RNR are shown cranially to the stenotic level, but they can appear caudally or both [13–15]. Patients who show RNR on preoperative MRI have a longer history, more severe symptoms, a smaller DCSA, and a lower postoperative recovery rate than patients without RNR [16, 17]. Therefore, we considered RNR as a negative prognostic factor. It is of interest to evaluate which posture provides “RNR-free” time, and to what extent. This positional MRI study aimed to answer four questions:

1. Does the transition from the extended lumbar spinal canal in the STA to the inflected spinal canal in the FLEX affect the prevalence and direction of redundant nerve roots?
2. Does the stenotic lumbar spine behave differently than the non-stenotic spine in terms of LL, LSC, and APD?
3. Does the change in body posture influence the APD of the stenotic level differently from that of the non-stenotic adjacent levels?
4. How reliable is the ASED-classification of RNR [18] when applied to the imaging of a 0.6-T MRI?

## Methods

### Study design

This study was a retrospective and observational with a repeated measures design. Files between June 2011 and May 2021 of the same Upright-MRI database were selected according to the following inclusion criteria: single-level degenerative cLSS causing RNR in STA and positional MRI study in at least two further postures (FLEX, SUP, and/or SIT) in T2-weighted (T2WI) modality. The control group consisted of individuals who underwent the investigation because of nonspecific lower back pain and had undergone a positional MRI scan as mentioned above. The exclusion criteria included single-level stenosis without evidence of RNR, two- or multi-level stenosis, previous surgery at the index level, previous fixation devices in the lumbar spine, and acute trauma. The files were anonymized for the purposes of this study.

The Ethics Committee of the Federal State of Hamburg does not require informed consent or approval for a retrospective observational study when the data is acquired, saved, and treated anonymously. The authors adhered to the Strengthening the Reporting of Observational Studies in Epidemiology (STROBE) guidelines [19].

### Study sample

Sixty-eight patients with single-level cLSS underwent the positional MRI (FONAR Upright™, 0.6 T, FONAR Corp.; Melville, NY 11747, USA) as they were surgical candidates. All patients underwent STA, FLEX (T2WI), and SIT (T1WI) examinations. Forty-one of them were additionally examined in SUP (T2WI, protocol 1). The remaining 27 patients were examined in SIT (T2WI, protocol 2) to shorten the investigation time due to claustrophobia or obesity-related issues. Patients in SUP position lay flat on their backs with a small pillow beneath the knees to relax the psoas muscle. Forty-five controls were investigated according to protocol one (Fig. 1). The whole cohort underwent SIT (T1WI) for completion of diagnostics.

### MR protocol

The standard imaging protocol included the following sequences:

- STA: Sag T2 FSE, TE 110 ms/TR 1140 ms, Matrix 480 × 480, FOV 330; Axi T2 FSE, TE 140 ms/TR 1440 ms, Matrix 512 × 448, FOV 240
- SUP: Sag T2 FSE, TE 132 ms/TR 1904 ms, Matrix 540 × 480, FOV 310
- SIT: Sag T2 FSE, TE 132 ms/TR 1904 ms, Matrix 540 × 480, FOV 310; Sag T1 SE, TE 20 ms/TR 500 ms, Matrix 540 × 480, FOV 310; Axi T2 FSE, TE 132 ms/TR 2167 ms, Matrix 420 × 360, FOV 180; Cor STIR, TE 100 ms/TR 2240 ms, Matrix 400 × 340, FOV 350
- FLEX: Sag T2 FSE, TE 132 ms/TR 1904 ms, Matrix 540 × 480, FOV 310

Mean scanning time was 45 min and slice thickness was 4 mm for each sequence.

### Assessment of the study variables

**Fig. 2:**
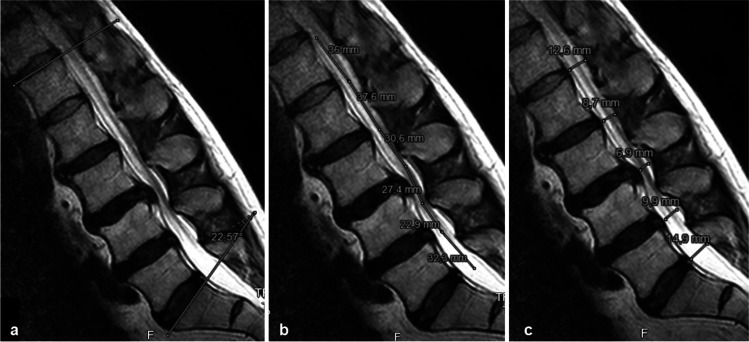
Parameters: a lumbar lordosis (LL): angle between the upper endplate L1 and the upper endplate S1. b Length of the spinal canal (LSC): sum of the segmental lengths parallel to the main bundle of cauda nerve roots between the upper endplate L1 and the upper endplate S1. c Anteroposterior diameter (APD): segmental sagittal diameter of the dural sac at the mid-disk level

**Fig. 3:**
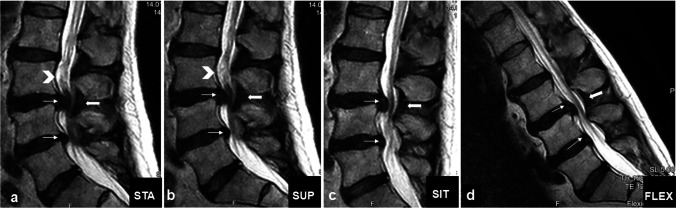
Clinical case: a 73-year-old man presented with neurogenic claudication. In the previous 3 weeks, he experienced exacerbation of leg pain and new hypesthesia in the lower limbs. a Standing: RNR (arrowhead) cranial to the pincer stenosis caused by the buckling of disks (slim arrows) and yellow ligament (thick arrow). b No relevant difference in supine posture was observed. c In neutral sitting partial flattening of disks and yellow ligament with increase in the anteroposterior diameters and resolution of RNR was observed. d Enlargement of the stenotic level and complete resolution of the RNR

Parameters LL, LSC, and APD L1-L5 are shown in Fig. 2. Instead of measuring along the posterior border of the vertebral bodies, LSC was intentionally measured among the cauda nerve roots, to monitor the change in their course caused by different postures. DCSA was measured on axial STA (T2WI) and SIT (T2WI) slices (Fig. 3). As per Schizas et al. [20], the Lausanne classification was used for qualitative assessment of stenosis severity. In grade B stenosis, cerebrospinal fluid is still visible around the CNR in the axial T2WI MRI image, unlike in grades C and D stenosis. For this study, grade B stenosis was defined as CSF + , and grades C and D were defined as CSF − . All measurements were performed jointly by three authors (LP, NA, KS) using the JiveX DICOM Viewer (VISUS Health IT, Bochum, Germany).

The ASED classification for RNR [18] was used for sagittal T2WI in the STA, SUP, SIT, and FLEX positions. The images were classified jointly by three authors (LP, KS, NA), and repeated 8 weeks later to circumvent any issue caused by inferior imaging quality of 0.6-T MRI compared with 3-T MRI. Discrepancies were resolved through consensus.

### Statistical analysis

Statistical analyses were performed using SPSS for Windows, version 24.0 (SPSS Inc., USA). Metric variables were presented as means and medians, and dispersion measures were presented as standard deviations and quartiles. Two-sided confidence intervals (CI) were set at 95%. Categorical or nominal data were reported as absolute or relative frequencies and analyzed using the chi-square test or Fisher’s exact test. When comparing two independent, normally distributed samples, the Student’s *t*-test was used. Paired *t*-test was used when the samples were dependent. Levene’s test was performed to check for homogeneity of variance. Welch’s *t*-test was performed in the absence of the latter. Two-sided significance tests were performed. A *p* value less than 0.05 was set as the criterion for statistical significance.

## Results

Patients and controls were comparable in terms of mean age (68 ± 10 years; *p* = 0.88) and sex distribution (31% vs. 42% women; *p* = 0.22). In Table 1, the changes in LL, LSC, and APD in the four body positions are compared between the patients and controls.

**Table 1:** Comparison of LL (°), LSC (mm), and APD (mm) between patients and controls in four different body postures and prevalence of RNR

Mean ± SD [95% CI]	STA—LL	SUP	SIT	FLEX
Patients	45.01 ± 10.97 [42.36, 47.67]	42.29 ± 9.37 [39.33, 45.25]	23.69 ± 10.92 [21.05, 26.33]	8.59 ± 7.96 [6.66, 10.52]
Controls	51.11 ± 11.33 [47.71, 54.52]	45.42 ± 9.75 [42.49, 48.35]	29.84 ± 13.93 [25.66, 34.03]	10.84 ± 11.86 [7.28, 14.41]
*p* value	**0.005**	0.134	**0.015**	0.228
Mean ± SD [95% CI]	STA—LSC	SUP	SIT	FLEX
Patients	156.37 ± 11.97 [153.47, 159.27]	157.90 ± 11.92 [154.14, 161.66]	163.53 ± 11.95 [160.64, 166.42]	168.82 ± 11.81 [165.96, 171.68]
Controls	153.33 ± 11.03 [150.02, 156.65]	155.89 ± 10.99 [152.59, 159.19]	162.47 ± 12.95 [158.58, 166.36]	169.02 ± 13.29 [165.03, 173.01]
*p* value	0.117	0.417	0.655	0.934
Mean ± SD [95% CI]	STA—APD	SUP	SIT	FLEX
Patients	10.95 ± 2.92 [10.24, 11.66]	10.90 ± 2.60 [10.07, 11.72]	11.45 ± 2.39 [10.78, 12.03]	12.81 ± 2.72 [12.15, 13.47]
Controls	12.63 ± 2.49 [11.88, 13.37]	12.99 ± 2.67 [12.19, 13.79]	12.81 ± 2.20 [12.15, 13.47]	14.14 ± 2.69 [13.33, 14.59]
*p* value	**0.002**	** < 0.001**	**0.003**	**0.012**
	STA—RNR	SUP—RNR	SIT—RNR	FLEX—RNR
%	100	49	26	4
[95% CI]	–	[32.88, 64.87]	[11.11, 46.28]	[0.92, 12.36]

*LL*, lumbar lordosis angle L1/S1; *LSC*, length spinal canal L1-S1; *APD L1-L5*, sum of the antero-posterior diameter at the mid-disk levels; Bold *p* < 0.05

### Prevalence and direction of RNR

**Fig. 4:**
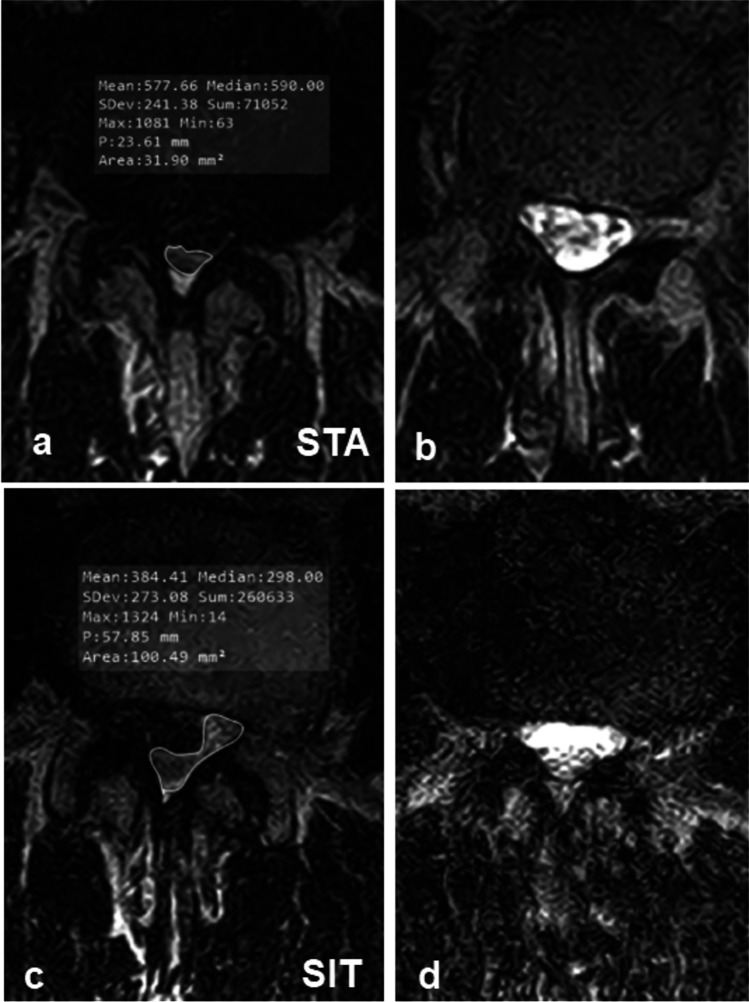
Same patient as in Fig. 1: dural cross-sectional area (DCSA) in standing position at the stenotic level (**a**) and 10-mm cranial (**b**) coiled and loop-shaped cauda nerve roots with positive sedimentation sign. **c** In sitting DCSA trebled, cauda nerve roots run perpendicular to the axial plane and the sedimentation sign became negative

Sixty-eight patients showed RNR in the STA. The first subgroup of 41 patients underwent a SUP examination. In this body posture, RNR persisted in 20 patients (49%). The second subgroup of 27 patients underwent SIT examination. In this body posture, RNR persisted in seven patients (26%). All patients underwent FLEX examination. In this body posture, RNR persisted in three patients (4%). Only the CSF + in STA variable predicted the resolution of RNR in SUP (*p* = 0.005) or SIT (*p* = 0.004), whereas the changes in other parameters such as LL (*p* = 0.21), LSC (*p* = 0.77), and APD (*p* = 0.91) did not. The location of the RNR at the stenotic level is shown in Fig. 4. Notably, in the three body postures subject to axial gravity (STA, SIT, and FLEX), the majority of RNR were located caudal to the stenotic level.

### LL

**Fig. 5:**
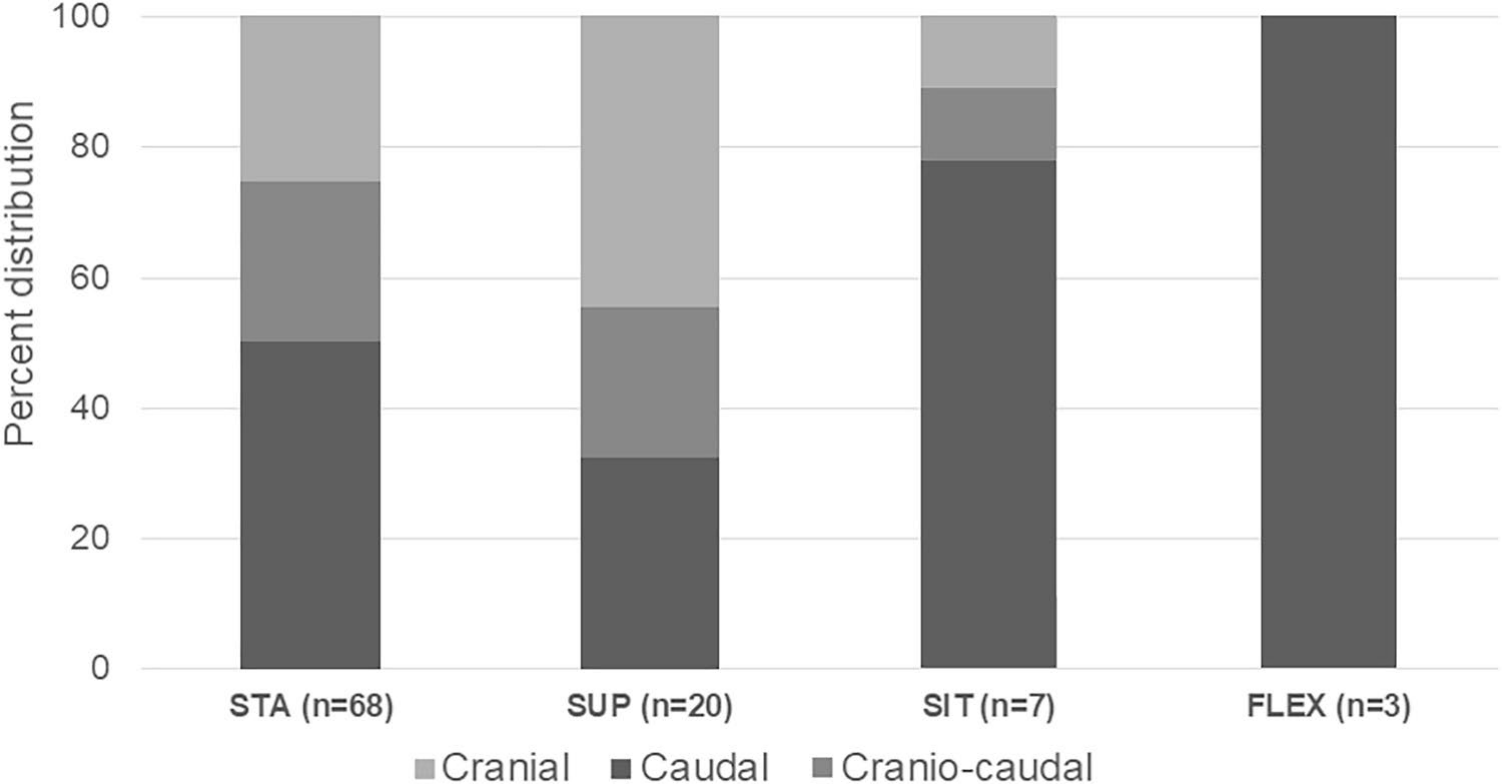
Percent distribution of RNR location to the stenotic level. Although the data of some subgroups is small, there is a trend for caudal location of RNR in body postures influenced by axial gravity

LL was significantly lower in patients in STA (*p* = 0.005) and SIT (*p* = 0.015) groups than in control group. A possible explanation for this is the antalgic slightly bent forward posture of the patients affected by cLSS. However, LL did not differ between patients in SUP (*p* = 0.134) and FLEX (*p* = 0.228) groups compared to control group. While the SUP position is “standardized” due to the use of a horizontal table, the FLEX seated position is actively maximized by the patients (Fig. 5).

In patients, STA-LL decreased to SUP-LL by 6%, to SIT-LL by 18%, and to FLEX-LL by 81%. In the controls, STA-LL decreased to SUP-LL by 11%, to SIT-LL by 42%, and to FLEX-LL by 79%. Thus, the significant difference in STA-LL between patients and controls (*p* = 0.005) was equalized in the FLEX group (*p* = 0.228).

### LSC

**Fig. 6:**
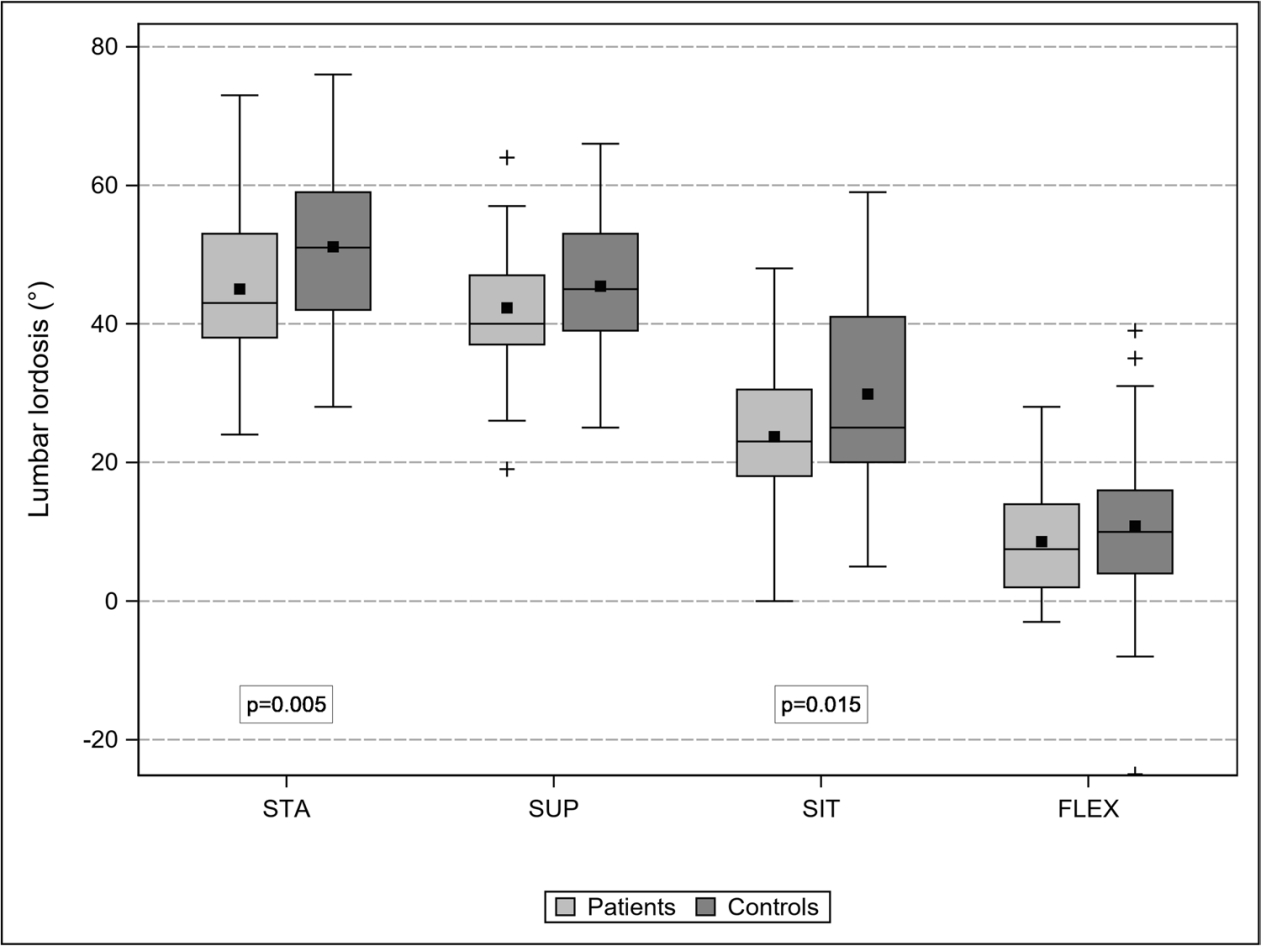
Comparison of the change of LL between patients and controls: progressive flattening from STA to FLEX in both cohorts. The difference is significant only in STA and SIT

The comparison of LSC in patients vs. controls in STA, SUP, SIT, and FLEX did not show any significant differences (Fig. 6).

LSC increased in patients from STA to SUP by 1%, SIT by 5%, and FLEX by 8%. LSC increased in controls from STA to SUP by 2%, SIT by 6%, and FLEX by 10%. Hence, the maximum LSC did not differ between the groups (*p* = 0.934).

### APD

**Fig. 7:**
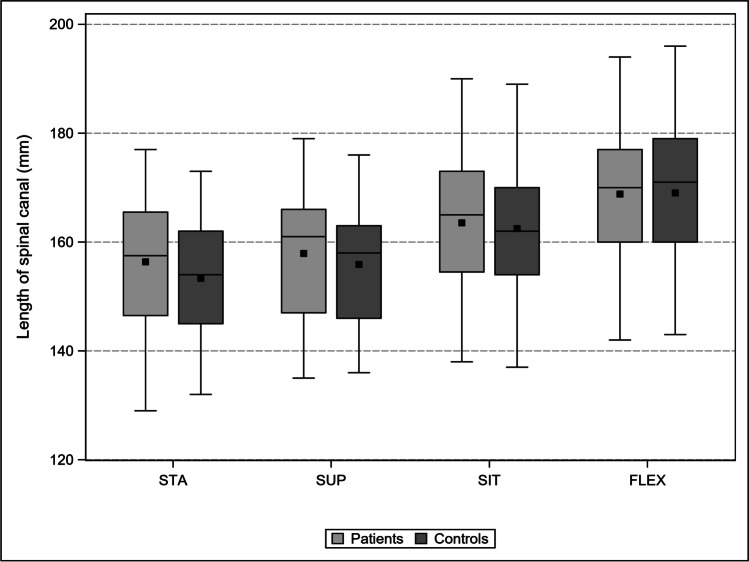
Comparison of lumbar spinal canal length (mm) changes between patients and controls: progressive and comparable lengthening from STA to FLEX

**Fig. 8:**
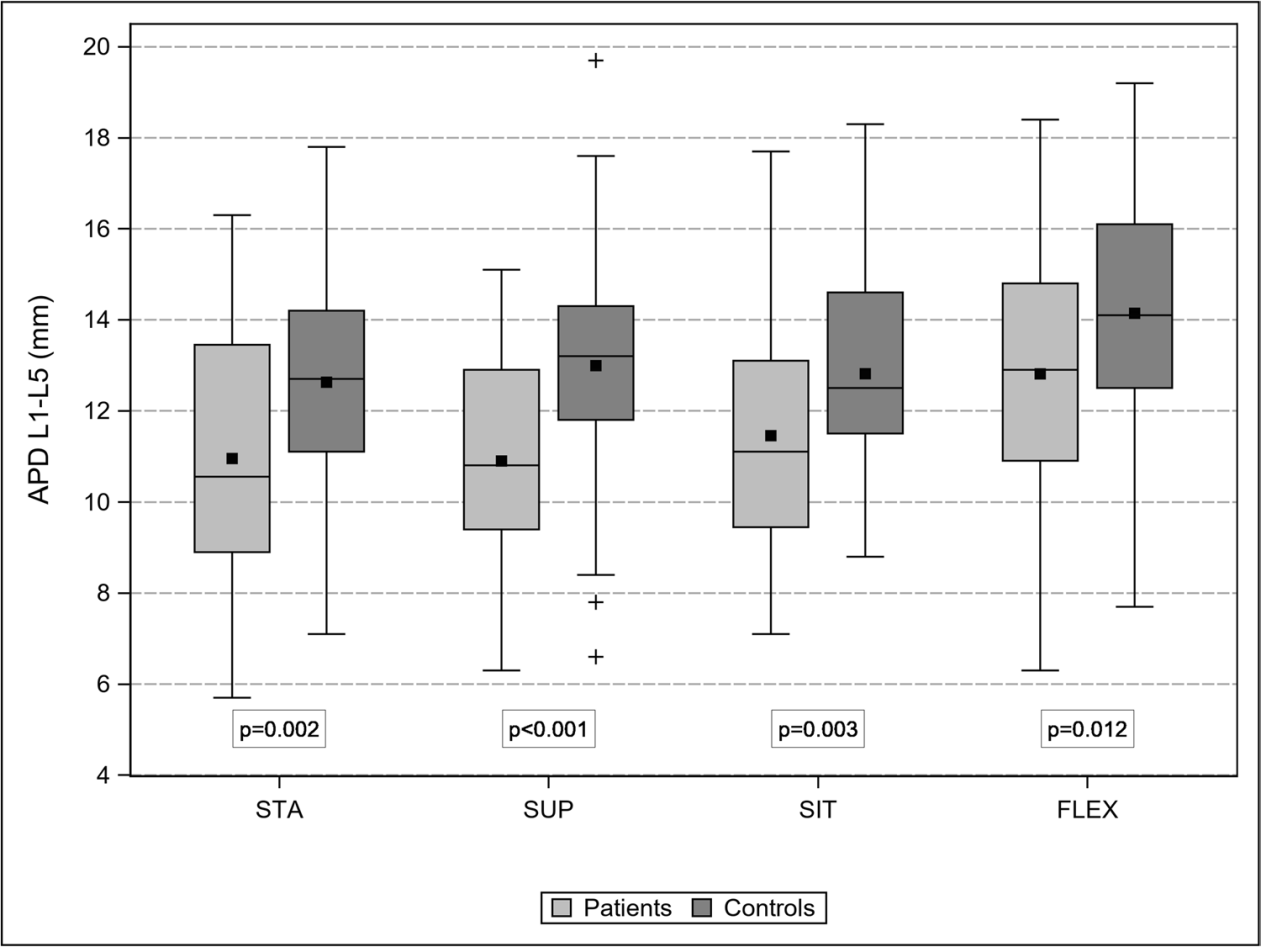
Comparison of the APD L1-L5 (mm) between patients and controls: the dynamic is comparable although the absolute values differ significantly

**Fig. 9:**
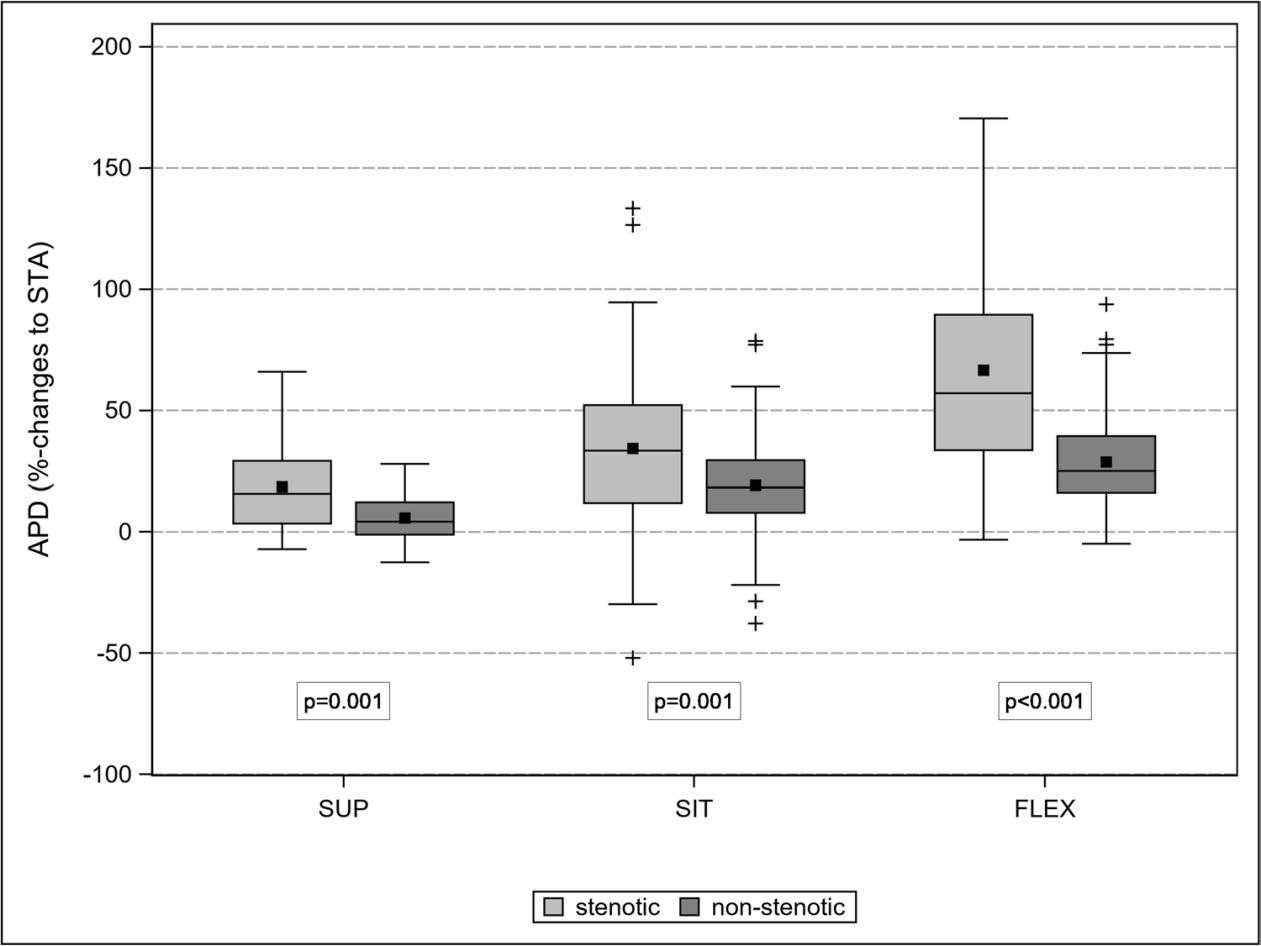
Comparison of the body posture related increase of APD between the stenotic level and the four non-stenotic levels. The disproportionate enlargement of the stenotic level gradually compensates its initial insufficient segmental intraspinal volume

APD L1-L5 in patients did not change substantially from STA to SUP (by − 0.4%), increased to SIT by 5%, and to FLEX by 17%. APD L1-L5 increased in controls by 3%, 1.4%, and 14%, respectively. In both groups, the spinal canal expanded with comparable dynamics (*p* = 0.78), although at a different capacitance (Fig. 7). Interestingly, the APD of the stenotic level increased from STA to SUP by 18% (*p* = 0.001), to SIT by 34% (*p* = 0.001), and to FLEX by 67% (*p* < 0.001) (Fig. 8). The corresponding values for the non-stenotic adjacent levels were 6%, 19%, and 29%, respectively. The prevalence of RNR dropped dramatically during the transition from the extended spinal canal in the STA to the flexed spinal canal in FLEX due to the disproportionate enlargement of the stenotic level, which almost doubled the expansion with each change in posture (Fig. 9).

### DCSA

The critical CNR cross-sectional area at the level L1/L2 was 77.4 ± 13.3 mm^2^ and decreased roughly by 10 mm^2^ at each further caudal level [21]. Schoenstroem et al. [22, 23] measured an in vitro pressure of 50 mmHg when the area was constricted to 62.2 ± 12.0 mm^2^. A further reduction of the area to 56.8 ± 10.05 mm^2^ (− 25%) doubled the pressure to 100 mmHg. These data show that the CNR cross-sectional area is crucial for functioning roots. In STA, the DCSA of the stenotic CSF + levels (101.60 ± 26.98 mm^2^) exceeded the threshold and caused “fluid-dynamic” RNR, possibly caused by the increased velocity of the CSF through the stenotic area, influence of gravity, increased drag force on the CNR, and post-stenotic drop of the hydrostatic pressure [24]. Conversely, the DCSA of CSF − levels (49.16 ± 21.02 mm^2^) fell below the threshold and caused “anatomical” RNR due to the mechanical constriction of CNR at the narrowest DCSA.

### ASED classification

The challenge of classifying RNR on MRI (0.6 T) was in selecting the most valid sagittal slice, especially in patients with coronal deformity of the lumbar spine. Therefore, consensus among the three raters was recorded. Eight weeks later, the rating process was repeated. The percentage agreement is presented in Table 2.

**Table 2:** Percent agreement of three raters in classifying RNR twice within 8 weeks

Body posture (No pat./RNR +)	RNR pos/neg	Allocation	Shape	Extension	Direction
STA (68/68)	100	95.6	91.2	94.1	85.3
SUP (41/19)	90.2	90.2	100	95.1	100
SIT (27/7)	92.6	92.6	100	92.6	100
FLEX (68/3)	100	100	100	100	100

## Discussion

RNR are rarely associated in conventional supine MRI with disk herniation or tumor [25, 26], but it has been reported in 33–43% of patients with spinal canal stenosis [12, 27, 28]. Coiled,elongated, and thickened CNR is clinically relevant as a negative prognostic factor [12, 17, 28]. Patients who show RNR on preoperative MRI have a longer history, more severe symptoms, and a lower postoperative recovery rate than those without RNR [16 17]. The current mechanical squeeze theory of RNR dates to 1992, when Suzuki et al. suggested that at the stenotic spine level, the pulsatile movements of the CNR are restricted or blocked. Repeated flexion of the lumbar spine stretches and elongates the nerve roots, and spine extension causes tortuosity, redundancy, and coiling. A more common location of RNR above the stenotic level seems to support this mechanism [12]. Current knowledge about RNR has mostly been forged by anatomical, myelographic, and supine MRI studies. An earlier positional MRI study [7] on RNR comparing the STA and FLEX positions, but lacking the supine position, showed that the prevalence and location of RNR depends on the body posture. This concept of highly dynamic RNR focuses on the fluid-dynamic aspects beyond the mechanical constriction of the CNR. The present study evaluated a larger number of patients, completed the MRI examination in the supine position, with a cohort of age- and sex-matched control patients suffering from back pain.

First, our study shows that the transition from the extended lumbar spinal canal in the STA to the inflected spinal canal in FLEX affects the prevalence and direction of redundant nerve roots. As a rule of thumb, each postural change in the STA sequence halves the prevalence of RNR. Furthermore, the resolution of RNR from the STA to the SUP correlates strongly with the severity of stenosis. In STA, a mild anatomical stenosis may cause CSF flow turbulence that generates RNR mostly below the stenotic level but resolves in SUP. In severe cases, such as CSF − condition, stenosis traps the traversing CNR in STA as well as in SUP. Additionally, the prevalent location of RNR being caudal to the stenotic level in all body postures exposed to axial gravity (all postures except SUP) confirms the role of gravity on CSF fluid movements.

Second, we find that the stenotic lumbar spine does not behave differently from the non-stenotic spine in terms of LL, LSC, and APD during posture changes. Although the absolute figures are lower in the former, the stenotic spine behaves comparably to the non-stenotic spine.

Third, changing body posture influences the APD of the stenotic level differently from that of the non-stenotic adjacent levels. The stenotic level disproportionally increased the intradural capacitance when compared with the non-stenotic levels in the same spine. Combined stretching of the buckling yellow ligament, usually remarkably thickened, and the flattening of the posterior disk, usually remarkably bulging, during progressive flexion of the spine could anatomically explain the difference between stenotic and non-stenotic levels. More trivial, but noteworthy, is the logic of small numbers: the 100% increase of a stenotic APD from 3 to 6 mm is functionally more relevant than the 27% increase of a non-stenotic APD from 11 to 14 mm at the adjacent level, although in both cases, the difference is 3 mm. In contrast, if in the MRI-SUP investigation of a patient affected by cLSS, stenosis should be maximized to resemble the findings in STA, the comfortable psoas-relaxed position should be substituted with a combination of straightening legs (anterior pelvic tilt) and placing a lumbar pillow underneath the back. Madsen et al. showed that this positioning best resembles STA [29]. We agree with this pragmatic alternative to the STA investigation, which is not currently implemented. However, we are aware of two limitations of this alternative: the influence of gravity is eliminated, and the horizontal table restricts the antalgic posture while standing.

Lastly, the present study tested the reliability of the ASED classification of RNR. The classification is intended to facilitate communication between radiologists and clinicians by referring to imaging details with clinical prognostic relevance. The strong agreement rate confirmed the reliability of the classification, even with reasonable, but not excellent, imaging quality.

The retrospective design of this study was biased by its intrinsic limitations. The selective inclusion criteria led to a relatively small number of patients, even when recruited over a decade. Furthermore, the partial change in the imaging protocol during this time generated two even smaller subgroups (SUP and SIT). Some studies indicate that DCSA measurement is more sensitive than APD for detecting changes in the width of the spinal canal. In this study, the DCSA was measured only in two postures due to time constraints. On CT, the correlation between the DCSA and APD is strong [3]. Nevertheless, our findings support reports in the literature that RNR are a sign of advanced and clinically relevant lumbar stenosis. The sequential decrease of the prevalence of RNR in the four body postures mirrors the spontaneous choice of the patients affected by cLSS during daytime (squatting, flexed sitting, and walking) and at night (sleeping in fetal position, unpublished results) aiming to increase their “RNR-free” time. The temporary effectiveness of flexion exercises or the segmental kyphosis due to interspinous devices indicates similar outcomes. The high dynamics of RNR, which may be based on fluid-dynamic aspects, are the topic of an ongoing study to further decipher details of RNR.

## Conclusions

Positional MRI shows that the prevalence of RNR in conventional supine MRI is underestimated by half. Posture changes modify LL, LSC, and APD similarly in patients with cLSS stenotic spines as in patients with non-stenotic low back pain. Stenotic levels compensate for insufficient intradural volume with disproportionate enlargement.

## Abbreviations

ASED: Allocation-Shape-Extension-Direction—classification of RNR
APD: Segmental antero-posterior diameter
APD L1-L5: Cumulative antero-posterior diameter L1-L5
CNR: Cauda nerve roots
cLSS: Central lumbar spinal stenosis
DCSA: Dural cross-sectional area
FSE: Fast spin echo
FLEX: Flexed seated position
LL: Lumbar lordosis
LSC: Length of the spinal canal
RNR: Redundant nerve roots
SE: Spin echo
SIT: Neutral seated position
STA: Standing position
SUP: Supine position
TE: Echo time
TR: Repetition time
T1WI: T1-weighted images
T2WI: T2-weighted images
